# Genome-wide association and evolutionary analyses reveal the formation of swine facial wrinkles in Chinese Erhualian pigs

**DOI:** 10.18632/aging.102078

**Published:** 2019-07-15

**Authors:** Tao Huang, Mingpeng Zhang, Guorong Yan, Xiaochang Huang, Hao Chen, Liyu Zhou, Wenjiang Deng, Zhen Zhang, Hengqing Qiu, Huashui Ai, Lusheng Huang

**Affiliations:** 1 State Key Laboratory of Pig Genetic Improvement and Production Technology, Jiangxi Agricultural University, Nanchang, P.R. China

**Keywords:** pig, facial wrinkles, GWAS, evolutionary analyses, artificial selection

## Abstract

Wrinkles are uneven concave-convex folds, ridges or creases in skin. Facial wrinkles appear in head, typically increasing along with aging. However in several Chinese indigenous pigs, such as Erhualian pigs, rich facial wrinkles have been generated during the growth stages as one of their breed characteristics. To investigate the genetic basis underlying the development of swine facial wrinkles, we estimated the folding extent of facial wrinkles in a herd of Erhualian pigs (n=332), and then conducted genome-wide association studies and multi-trait meta-analysis for facial wrinkles using 60K porcine chips. We found that facial wrinkles had high heritability estimates of ~0.7 in Erhualian pigs. Notably, only one genome-wide significant QTL was detected at 34.8 Mb on porcine chromosome 7. The most significant SNP rs80983858 located at the 3255-bp downstream of candidate gene *GRM4*, and the G allele was of benefit to increase facial wrinkles. Evolutionary and selection analyses suggested that the haplotypes containing G allele were under artificial selection, which was consistent with local animal sacrificial custom praying for longevity. Our findings made important clues for further deciphering the molecular mechanism of swine facial wrinkles formation, and shed light on the research of skin wrinkle development in human or other mammals.

## INTRODUCTION

The skin is the largest organ of the integumentary system in mammals and serves as the first line of defense from external factors. Usually skin wrinkles emerge and increase along with the aging processes, and extensive skin wrinkles exist in elder mammals, especially for facial wrinkles. According to histological differences and possible causes, skin wrinkles were typically classified into five categories: atrophic wrinkles, elastotic wrinkles, expressional wrinkles, gravitational wrinkles and sleep wrinkles, respectively [1–4]. It has been reported that many intrinsic and extrinsic factors contributed to skin wrinkle development and formation [4, 5], which included slower epidermal turnover, increased skin fragility, decreased nutrients transfer between skin layers, loss of sebaceous glands and reduced oil production in skin, loss of fat support and less padding of fat tissue under skin, ultraviolet oxidative damage, harsh weather and pollution, and repeated skin movement.

In human, the extent of facial wrinkles directly indicated the age status and the spiritual condition (Supplementary Figure 1A). Smooth and lustrous skin represents health, beauty and youth. Many efforts have been made to postpone the time of wrinkle generation and reduce the puckering level of facial wrinkles. However in some mammals, heavy skin wrinkles developed along with the whole life stages and were always treated as their specific physical features. For instance, when compared with Chinese Tianyuan dogs, Chinese Shar-Pei dogs have much more heavy skin wrinkles (Supplementary Figure 1B); and compared with Chinese Lihua cats, Canadian Sphynx hairless cats also appear with richer wrinkles (Supplementary Figure 1C). Similarly, some Chinese indigenous pigs show obvious wrinkle appearance in the front head when they are young or at their growth stages. For examples, Chinese Erhualian (Supplementary Figure 1D), Meishan and Neijiang pigs have deep facial wrinkles when they are reaching sexual maturity [6]; on the contrary, European pigs, like Large white (Supplementary Figure 1D), Landrace and Duroc, have few facial wrinkles.

Erhualian, one of Chinese Taihu pig breeds, distributes mainly in the lower Yangtze River Basin in East China. It is globally famous for its highest reproduction performance with average litter size of more than 16 piglets [6]. Erhualian pigs appear with unusually heavy facial wrinkles and large floppy ears, which are their typical breed characters [7, 8]. In the present study, we employed two methods to estimate the folding extent of facial wrinkles in a herd of 332 Chinese Erhualian pigs, and we genotyped these pigs using the Illumina Porcine SNP60K Beadchip arrays, as well as their 9 sire parents. Then we performed genome-wide association studies, multi-traits meta-analysis and gene association analysis for facial wrinkles in this pig population. Furthermore, we investigated whether the target region harboring the major QTL was under artificial selection in the Erhualian pigs or not. The aim of this study is to uncover genetic loci associated with swine facial wrinkles. It might also help us to better understand the underlying molecular mechanisms of wrinkle formation in other mammals.

## RESULTS

### Descriptive statistics of phenotypic traits

In this study, we measured facial wrinkles using two different methods in a herd of Erhualian pigs involving 332 individuals. Descriptive statistics for both facial wrinkle phenotypes were shown in Table 1. The averages of wrinkle pixel ratio and wrinkle score were 0.09 ± 0.03 and 3.20 ± 1.04, respectively. The wrinkle pixel ratio ranged from 0.03 to 0.15, while the wrinkle score from 1 to 5. The two wrinkle phenotypes varied widely with the coefficients of 30.2% and 32.6%, respectively, which indicated that large divergence existed in the Erhualian pig population for the traits of facial wrinkles. And both phenotypes of facial wrinkles shared close relationship with a correlation coefficient of 0.777 (*P* < 0.01).

**Table 1:** Descriptive statistics for both facial wrinkle traits in Erhualian pigs.

**Trait**	**N_Ind_^1^**	**Range**	**Mean (SE)**	**CV**	***h*^2^**	**Cor^2^**
Wrinkle pixel ratio	332	0.03 - 0.15	0.09 (0.003)	30.15%	0.681	0.777
Wrinkle score	332	1 - 5	3.20 (1.04)	32.63%	0.737	

^1^N_Ind_ = the number of pigs;

^2^Cor = the correlation between wrinkle pixel ratio and wrinkle score.

To estimate sex effect on facial wrinkles, we classed Erhualian pigs into male and female groups. The average of the wrinkle pixel ratio were 0.08 and 0.09 in males and females, while the average of the wrinkle score were 3.06 and 3.35 in males and females, respectively (Supplementary Figure 2). The folding extent of facial wrinkles in females was slightly heavier than males (*P* = 1.87×10^-3^ and *P* = 9.93×10^-3^). Thus the gender slightly affects facial wrinkles in the Erhualian population, and sows generally have more facial wrinkles than boars.

### Heritability estimation

The heritability estimates of facial wrinkles were high, 0.68 for wrinkle pixel ratio and 0.74 for wrinkle score (Table 1), which indicated that the trait of facial wrinkles in the Erhualian pigs was highly heritable and might be determined by a remarkable genetic effect.

### Single-trait GWAS and multi-trait meta-analysis

In the 60K SNP data, no individual was removed due to low genotyping rate of less than 0.05, while 955 SNPs were excluded for high missing rate of more than 10% and 24,897 SNPs were removed for low minor allele frequency of less than 0.01. Finally, 35,977 SNPs and 332 individuals were kept for the next association analyses.

**Figure 1:**
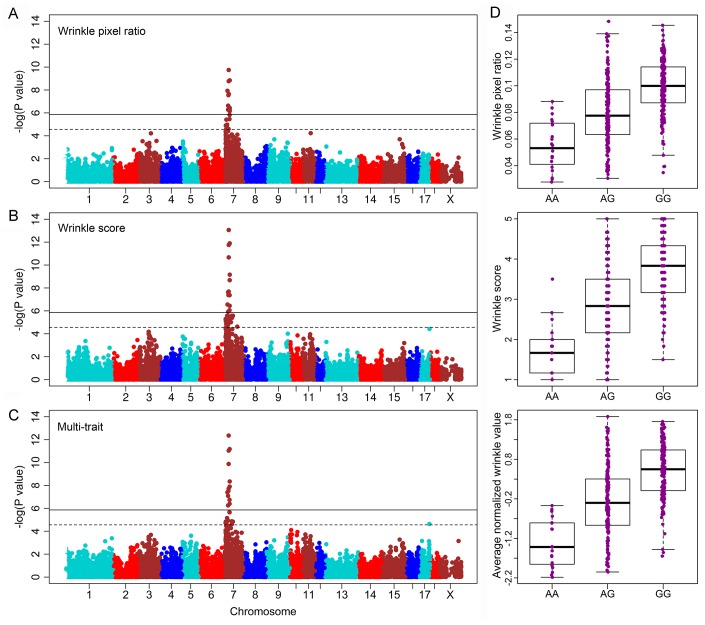
**Manhattan plots for QTL affecting facial wrinkles in Erhualian pigs.** (**A**) Genome-wide association study (GWAS) plot for wrinkle pixel ratio. (**B**) GWAS plot for wrinkle score. (**C**) Meta-trait GWAS plot for facial wrinkles. (**D**) Boxplot for the significant SNPs grouped by different genotypes. The dashed line delineates the suggestive significance threshold (*P* = 2.78 × 10^-5^); the solid line delineates the genome-wide significant threshold (*P* = 1.39 × 10^-6^).

Single-trait genome-wide association analyses were performed for both wrinkle phenotypes in the Erhualian pig population. To assess the plausibly of our observed data, we performed the “Q-Q” plot analysis for both wrinkle traits. The results showed that the distribution of observed *P*-values deviated from expected *P*-values in the end (Supplementary Figure 3). And the inflation factors (λ) of the GWAS for both traits are 1.017 and 1.023, suggesting that our GWAS results were reasonable. In the single-trait GWAS, we identified only one major QTL at 34.8 Mb on SSC7 for swine facial wrinkles, which contained 13 and 14 significant SNPs associated with wrinkle pixel ratio (Figure 1A) and wrinkle score (Figure 1B), respectively. The most significant SNP for both traits was same, rs80983858, which located at the 3,255 bp downstream of gene *GMR4*. It explained 21.80% of phenotypic variation for wrinkle pixel ratio and 31.3% for wrinkle score (Table 2).

**Table 2:** The information of most significant SNPs associated with facial wrinkle traits.

**Trait**	**Chr**	**N_snp_**	**Top SNP**	**Pos(bp)**	**Nearest Gene^1^**	**Distance (bp)^2^**	**Alleles**	**Effect**	***P*-value**	**Var(%)**
Wrinkle pixel ratio	7	13	rs80983858	34835986	*GRM4*	3255	G/A	-0.015	1.80×10^-10^	21.80%
Wrinkle score	7	14	rs80983858	34835986	*GRM4*	3255	G/A	-0.755	8.75×10^-14^	31.30%
Multi-trait	7	13	rs80983858	34835986	*GRM4*	3255	G/A	-0.755	4.45×10^-13^	

^1^Nearest gene = the gene on or nearest to the most significant SNP.

^2^Distance = the distance from the most significant SNP to the nearest gene; negative value indicates the SNP is on the upstream to the gene, positive value indicates the SNP is on the downstream region.

The two traits used for facial wrinkles measurements are highly correlated (Table 1), but they had different focuses: the pixel ratio mainly focused at the heavy wrinkle points, and the wrinkle score laid emphasis on the overall appearance. To validate the results of the single-trait GWASs and possibly increase the QTL power, we performed a two-trait meta-analysis. The two-trait meta-analysis was conducted based on the SNP effects from the single-trait GWAS. The QTL at 34.8 Mb on SSC7 was convinced with 13 significant SNPs passing over the suggestive significance threshold (1.39× 10^−6^), in which the most significant SNP was also rs80983858 with P = 4.45 × 10^-13^ (Figure 1C and Table 2). The effect of G allele is benefit to increase facial wrinkles (Figure 1D).

### Genome-wide gene association analysis

Genome-wide gene association analyses were performed for two phenotypes of facial wrinkles using MAGMA software in the Erhualian pig population. We identified only one major significant associated region at 34.8 Mb on SSC7 (Supplementary Figure 4), which was consistent with the result of SNPs-based GWAS. The top 10 significant genes for facial wrinkle score were *GRM4*, *DST*, *ZNF451*, *MLIP*, *CDKAL1*, *PRIM2*, *PRL*, *RNF144B*, *LOC100513868* and *FAM83B* (Supplementary Figure 4A); and the top 10 genes for wrinkle pixel ratio were *ZNF451*, *GRM4*, *MLIP*, *PRIM2*, *REC114*, *PRL*, *DST*, *TTC6*, *CDKAL1* and *STXBP6* (Supplementary Figure 4B). Among these genes, *GRM4*, *DST*, *ZNF451* and *MLIP* were strong candidate genes affecting the formation of swine facial wrinkles.

### Evolutionary and selection analyses

**Figure 2:**
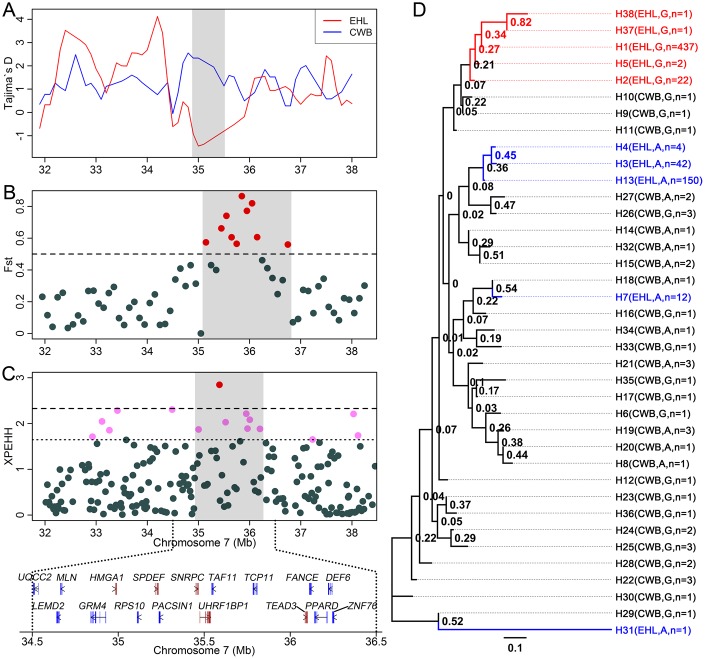
**Evolutionary and selection analyses on the candidate region affecting swine facial wrinkles using the 60K SNP dataset.** (**A**) Tajima’s D tests. (**B**) Population differentiation analysis between Erhualian pigs and Chinese wild boars. (**C**) XPEHH score estimation. (**D**) Maximum likelihood tree of the haplotypes on the candidate region.

To determine whether the candidate region affecting swine facial wrinkles was under selection or not, we have carried out three analyses on this region, including Tajima’s D test, genetic differentiation estimate of F_ST_ and calculation of XPEHH score. First, we merged the present 60K SNP dataset of 332 Erhualian pigs with previous 60K SNP dataset of 21 Chinese wild boars [9, 10]. We performed Tajima’s D test on the candidate region for Erhualian pigs and Chinese wild boars. Low Tajima’s D values less than -1 were detected in the Erhualian pigs but not in Chinese wild boars (Figure 2A), which suggested that this region was under selection in Erhualian pigs. We also found the region centered on 35.8 Mb ranging from 35.1 Mb to 36.8 Mb was highly differentiated between Erhualian pigs and Wild boars (Figure 2B). And high XPEHH scores were detected in this candidate region (Figure 2C). To validate the above results, we further performed these analyses on this region using our previous whole-genome sequencing dataset of Chinese Erhualian pigs [11, 12] and Chinese wild boars [12]. On the candidate region from 34.7 Mb to 36.2 Mb, the Tajima’s D values less than -1 were detected in the Erhualian pigs but not in Chinese wild boars (Supplementary Figure 5A). Meanwhile, high F_ST_ values (more than 0.15) and large XPEHH scores (more than 0.9) between Erhualian pigs and Chinese wild boars were observed on the target candidate region (Supplementary Figure 5B and 5C). These results strongly supported that the candidate region affecting the formation of facial wrinkles was under positive selection.

In addition, we found that the haplotypes containing G allele, benefiting to wrinkle formation in Erhualian pigs, were clustered into a single branch (Figure 2D). This result further suggested that the haplotypes with favorable wrinkle formation were under selection in Chinese Erhualian pigs.

## DISCUSSION

Naturally and generally, skin wrinkles rarely appear in the young or the growing individuals, especially for heavy facial wrinkles. Skin wrinkles usually increase along with age after adulthood. In human, there were few cases of heavy skin wrinkles in young people, but there are still some cases. For example in 2015, Chinese Chongqing Evening News has reported a 30-year-old patient, named Taiping Yuan, suffering heavy facial wrinkles (http://health.people.com.cn/n/2015/0902/c398004-27539627.html). However in some mammals, heavy winkles occur in young-age animals, like Chinese Shar-Pei dogs, Canadian Sphynx hairless cats and several Chinese indigenous pigs. What genetic factors resulted in breaking the natural rule of “young animals few wrinkles” in these mammals? The underlying genetic mechanism was worth paying extensive attention to deep exploration.

For skin wrinkles of Chinese Shar-Pei dogs, several great works have been previously done. Akey et al. (2010) reported that regulatory variation in *HAS2* gene was significantly associated with skin wrinkling in the Shar-Pei [13]. Further, Olsson et al., (2011) identified a 16.1 kb unstable duplication at the ~350 kb upstream region of *HAS2* gene predisposing to the breed-defining skin phenotype of heavy skin wrinkles [14]. However, Metzger and Distl (2014) doubted the association between wrinkled skin types and copy number variations of the upstream duplication, and proposed that the development of wrinkles together with familial Shar-Pei fever might underlie complex genetic mechanisms that need to be further unraveled [15].

As for the genetic study on swine skin wrinkles, Rothschild has conducted a pilot work to investigate the possible genetic wrinkle factors in pigs [16]. He has identified a 3.3-kb BglI fragment associated with swine excessive wrinkling in the F_1_ and F_2_ generations crossing by Meishan pigs and US pigs. Both Meishan and Erhualian pigs belong to Chinese Taihu pig breeds. In our present study, we employed a herd of Chinese Erhualian pigs as experimental materials; we developed two methods to estimate the folding extent of swine facial wrinkles. Then we performed genome-wide association studies, multi-traits meta-analysis and gene association analysis for facial wrinkles in this pig population. Only one genome-wide significant QTL for swine facial wrinkles was detected at 34.8 Mb on SSC7. The most significant SNP (rs80983858) located at the 3255-bp downstream of positional candidate gene *GRM4*, and the G allele was of benefit to increase facial wrinkles. Previously at around 34.8 Mb on SSC7, we have also identified QTLs affecting ear size [7], meat moisture content [17], skin thickness [18], fatness deposition [19], the weight of internal organs [12], limb bone lengths [20], head weight [21], and carcass length [22] in divergent pig populations, which indicated that this candidate region plays important roles in the development of many traits in pigs. This region is a gene-rich region, for example, there were 35 protein-coding genes from 34.0 to 35.5 Mb on SSC7, which suggested that the biological functions of this locus would be very important and complicate. This fact also makes it more difficult for us to identify causal genes for swine facial wrinkles, and the causal gene or causal mutation for facial wrinkles should be further exploration by the next biological experiments.

It is worth mentioning that the QTL for swine facial wrinkles in the Erhualian pigs is overlapped with the one for skin thickness [18] and ear size [7] in the Erhualian × White Duroc intercross pigs. The G allele of top significant SNP has the effect of increasing skin folds; it has also increasing effect on ear size and skin thickness. Therefore, we hypothesized that the extent of facial wrinkles were positively associated with skin thickening or skin proliferation.

In the candidate region, *GRM4, DST, ZNF451* and *MLIP* were top significant genes associated with swine facial wrinkles. *GRM4* is a family of G protein-coupled receptors that encode the group III metabotropic glutamate receptor 4, and plays roles in intracellular signaling and inhibition of the cyclic AMP signaling cascade [23, 24]. Several previous studies suggested that *GRM4* gene polymorphism was associated with susceptibility and prognosis of osteosarcoma in human [25–27]. Additionally, cyclic AMP was a key messenger in the regulation of skin pigmentation [28]; inhibitory effects of cyclic AMP elevating agents on lipopolysaccharide induced microvascular permeability change in mouse skin [29]. *DST* (Dystonin) gene encodes a member of the plakin protein family of adhesion junction plaque proteins. Interestingly, analysis of the human tissue-specific expression showed that *DST* gene was highest expressed in skin tissues [30]. In human, homozygous nonsense *DST* mutations affecting BPAG1e resulted in epidermolysis bullosa variant [31]. It was also reported that mice defective for this gene showed skin blistering and neurodegeneration [32]. *ZNF451* (Zinc Finger Protein 451) is a protein-coding gene associated with motion sickness, and most abundantly expressed in bone marrow and testis. *MLIP* (Muscular LMNA Interacting Protein) encodes alternatively spliced variants (23-57 kDa) and expressed most abundantly in heart, skeletal, and smooth muscle. To our knowledge, hitherto there are no studies to report *ZNF451 and MLIP genes* participating in the biological processes related to skin development. We speculated that possibly due to the hitchhiking effect of the causal gene, these two genes were associated with facial wrinkles in our present study.

Erhualian pigs belong to the so-called Taihu breed with high reproduction performance in the lower Yangtze River Basin in East China [6]. Erhualian pigs appear with unusually heavy facial wrinkles and large floppy ears, which are their typical breed characters [7, 8].

In ancient China, animal sacrifice was the ritual killing of an animal as part of a religion. During the animal sacrifice, people preferred to put the wrinkled swine head on the sacrifice desktop as a necessary ritual item to pray longevity, because wrinkled swine head looks like “longevity” on the head. Traditional Chinese character of “longevity” has complicated strokes, and ancient people would like to post the scroll consisting of hundred forms of the character of “longevity” in an important position of living or meeting room. Heavier wrinkled swine head indicates more “longevity”. So during the animal sacrifice, people preferred to put heavy wrinkled swine head on the sacrifice desktop as necessary ritual item. When raising pigs, people tend to select and keep heavy wrinkled pigs in the farm. Historical document, “the traditional Chinese new year chants” written by Guren Wu (Qing Dynasty), have recorded that people living in the region of Yangzi River basin in the Qing Dynasty preferred to select wrinkled swine head in the animal sacrifice, and more facial wrinkles in swine head more longevity. Our results suggested that the haplotypes with favorable wrinkle formation were under selection in Chinese Erhualian pigs, which was consistent with the history of animal sacrifice in the region of Yangzi River basin.

## MATERIALS AND METHODS

### Ethics statement

All procedures used for this study and involving animals are in compliance with guidelines for the care and utility of experimental animals established by the Ministry of Agriculture of China. The ethics committee of Jiangxi Agricultural University specifically approved this study.

### Experimental animals

A herd of Chinese Erhualian pigs (n=332) were used as experimental animals as described previously [33]. Briefly, the Erhualian pigs at the age of 80 ± 3 days from nine sires and 52 dams were bought from the national conservation farm for the Erhualian breed in Changzhou, Jiangsu province, which covered all sire lines and most of dam lines raised in the conservation farm. All piglets were raised twice per day with a corn- and soybean-based diet containing 16% crude protein, 3100 kJ digestive energy and 0.78% lysine at a farm in Nanchang, Jiangxi province, and with free access to water. Male pigs were castrated at the age of 60 days. All pigs including 168 males and 164 females were uniformly slaughtered at 300 ± 3 days at an abattoir for trait measurements.

### Phenotype measurements

After slaughter, swine heads were cut off at the joint between the first cervical vertebra and occipital bone. Front head picture of each individual was taken under same light condition using the camera of Xiaomi mobile phone Mi 3. We applied two methods to estimate the folding extent of facial wrinkles for all the experimental animals.

For the first method, wrinkle pixel ratio was calculated by comparing the sketch area of facial wrinkles to the whole area of facial contour with Photoshop software (Supplementary Figure 6A). First, each photo of the front head was imported into Photoshop software and was trimmed to remain the whole face by crop tool. Then we regulated the size of trimmed figure to fit a blank image with width size of 4 cm (472 pixels) and height size of 6 cm (709 pixels) without changing the width-to-length ratio by rectangular marquee tool and free transform tool. The images of 64 individuals arranged by 8 × 8 were placed on a 32 × 48 cm^2^ canvas, and then were printed on A4 paper with default option of "Scale to Fit Media". Their facial wrinkles and peripheral facial contours were traced on translucent copy paper over the printed images using HB pencil. The traced wrinkles and peripheral facial contours were scanned and reimported into Photoshop software. The area pixels of the traced facial wrinkles (TFW) and blank facial contours (BFC) were calculated by Photoshop software. Wrinkle pixel ratio was considered as TFW/BFC, representing one phenotype for the folding extent of facial wrinkles.

For the secondary method, we established a scoring criteria for swine facial wrinkles from 1 to 5, in which 1 represents minimum folding extent, 5 represents maximum folding extent (Supplementary Figure 6B). Three volunteers were convened to score the facial wrinkles in all experimental pigs at the scoring step of 0.5 according to the scoring criteria. The three volunteers were trained to read the score according to the scoring criteria of swine facial wrinkles before their formal scoring. If the score difference for a same sample was larger than 1 among the three independent volunteers, this sample would be selected out and independently rescored based on the scoring criteria by three volunteers until the difference was equal to or smaller than 1. Finally, the average values of wrinkle scores determined by these three volunteers were calculated and treated as another phenotype for the folding extent of facial wrinkles.

### SNP genotyping and quality control

Genomic DNA was extracted from ear tissues using a standard phenol/chloroform method. DNA concentration and quality were measured by a Nanodrop100 spectrophotometer (Thermo Fisher, USA) and agarose gel electrophoresis. All DNA samples were diluted to a final concentration of 50 ng/μl with ultraviolet absorption ratios of 260/280 > 1.8 and 260/230 > 2.0. Then these samples were genotyped for the Porcine SNP60 Beadchips on an iScan System (Illumina, USA) following the manufacturer’s protocol. Quality control of SNP genotype data was performed by PLINK (version 1.07). SNPs with a call rate < 90% and minor allele frequency (MAF) < 1% were removed from further analyses; individuals with missing genotypes > 10% or Mendelian errors > 5% were also excluded. Finally, 35,977 SNPs, including 477 SNPs from the X-chromosome were kept for GWAS analyses. Since male individual contains only one X chromosome, SNPs on the X-chromosome for males were treated as homozygous genotypes for further association analyses.

### Heritability estimates

Heritabilities of the measured traits were estimated based on the genotype data using the polygenic function implemented in R package of GenABEL [34]. A maximize polygenic model [35] was used as follows:

$$\text{logL}\;=\;\left(\text{1}/\text{2}\right)\left[{\text{log}}_{\text{e}}\left|\text{Σ}\right|\;+\;{\left(\text{y}\;-\;\text{μ}\right)}^{\text{T}}{\text{Σ}}^{-\text{1}}\left(\text{y}\;-\;\text{μ}\right)\right],$$

in which *y* indicates standardized phenotypic value, μ stands for the average standardized phenotypic value, Σ denotes the variance-covariance matrix, and Σ = AV_A_ + IV_R_. Here, A is the relationship matrix of kinship, which is estimated from genetic data; V_A_ is the additive genetic variance due to polygenes; I stands for the identity matrix; and V_R_ is the residual variance. Heritability estimates were calculated with the equation *h*^2^ = V_A_/(V_A_ + V_R_).

### Single-trait genome-wide association studies

Association between each SNP and phenotypes of facial wrinkles in the population was evaluated under a generalized linear mixed model using polygenic and *mmscore* function implemented in GenABEL package [34]. The statistic model is shown as follows:

y = Xβ + Sα + Zμ + e,

in which *y* is the vector of phenotypes; *β* is the estimator of fixed effects including sex and batch; *α* is the vector of SNP substitution effects; and *μ* is the random additive genetic effect following a multivariate normal distribution μ ~ *N*(0, *G*V_G_), in which *G* is the comparability kinship matrix between each individual estimated by whole-genome SNP as described in [36] and V_G_ is the polygenic additive variance; *X*, *S*, and *Z* are the incidence vectors (matrixes) for *β*, *α*, and *μ*, respectively; and *e* is a vector of residual effects normally distributed *N*(0, lV_E_), V_E_ is the residual variance.

Genome-wide significance thresholds were set as *P*_0.05_ = 0.05/N with Bonferroni adjustment, and the suggestive significance threshold was set to be 1/N, where N is the number of filtered SNPs in the data set. In this study, the thresholds were set as 1.39 × 10^-6^ (0.05/35977) and 2.78 × 10^-5^ (1/35977), respectively. The 2-LOD drop-off method was used to determine confidence intervals of the identified loci [37]. Percentage of variance (%) explained by each top significant SNP in the GWAS was calculated using the following formula:

$$\text{var}\;=\;\left[\left({\text{MS}}_{\text{reduce1}}\;-\;\;{\text{MS}}_{\text{full}}\right)/{\text{MS}}_{\text{reduce}}\right]\;\times \;\text{1}00,$$

in which MS_reduce1_, MS_full_, and MS_reduce_ indicate the mean square (MS) in the linear models including 3 effects (mean, sex and batch), 4 effects (mean, sex, batch and SNP), and only mean, respectively.

### Multi-trait meta-analysis

A Chi-square statistic was calculated for the importance of the effects of SNP_i_ (i=1, 2, 3, …, 35977) across both wrinkle phenotypes estimated in the present study, which approximately follows a Chi square distribution with 2 degrees of freedom. For each SNP, the multi-trait statistic was calculated by the formula [38]:

$$\chi_{multi-trait}^{2}\;=\;t_{i}^{'}\;V^{-1}\;t_{i}$$

where t_i_ is a 2×1 vector of the signed t values of the ith SNP for the two wrinkle traits, t’_i_ is the transpose of vector t_i_(1×2), *V*^-1^ is the inverse of 2×2 correlation matrix between two traits is the correlation over the 35,977 SNPs effects(signed t values) of the two traits.

### Genome-wide gene association analysis

The SNP-based *P* values from the single SNP-by-SNP GWAS were used as input for the gene-based analysis. We used all 16,852 Sus scrofa protein-coding genes from the NCBI gene definitions as the basis for a genome-wide gene association analysis (GWGAS) in MAGMA (http://ctg.cncr.nl/software/magma). After SNP annotation, there were 5,520 genes that were covered by at least one SNP. Gene association tests were performed taking LD between SNPs into account. We applied a stringent Bonferroni correction to account for multiple testing, setting the genome-wide threshold for significance at 9.06 × 10^−6^.

### Evolutionary analyses for the target region

The target region centering around the QTL from 32 to 38 Mb on SSC7 was chose for the evolutionary and selection analyses employing two datasets. The SNP data of 21 Chinese wild boars [9, 10] were included and merged together with the present data of Erhualian pigs to form the first 60K SNP dataset. Previous whole- genome sequencing data of 19 Erhualian pigs [11, 12] and 6 Chinese wild boars [12] with average depth of ~25X were combined together to form the second sequencing dataset. Genetic differentiation estimates of F_ST_ between Erhualian and wild boar populations were calculated as described in Weir’s paper [39] using the SNP dataset on the target region. Tajima’s D within both Erhualian and wild boar populations were estimated as described in Tajima’s paper [40]. The average F_ST_ and Tajima’s D were calculated in sliding windows of 200 kb with step size of 100 kb. Selection footprints on the target region were also detected by a between-population method of XPEHH implemented in Selscan software [41].

Phases of the 25 SNPs within the target region (from 34,634,619 to 35,422,882 bp on SSC7) were reconstructed using Phase v2.1.1 [42] with default options. Phased alleles were linked together to form haplotype sequences, which were used for reconstruction of neighbor-joining (NJ) phylogenetic trees by MEGA7 [43] with 1,000 times bootstrapping.

## Supplementary Material

Supplementary Figures
