# Supplementary material for: Genome-wide association and evolutionary analyses reveal the formation of swine facial wrinkles in Chinese Erhualian pigs
Source: Aging (Albany NY). 2019 Jul 15;11(13):4672–87. doi: 10.18632/aging.102078 (PMC6660038; doi:10.18632/aging.102078)
Supplement: Supplementary Figures [file aging-11-102078-s001.pdf]

SUPPLEMENTARY FIGURES

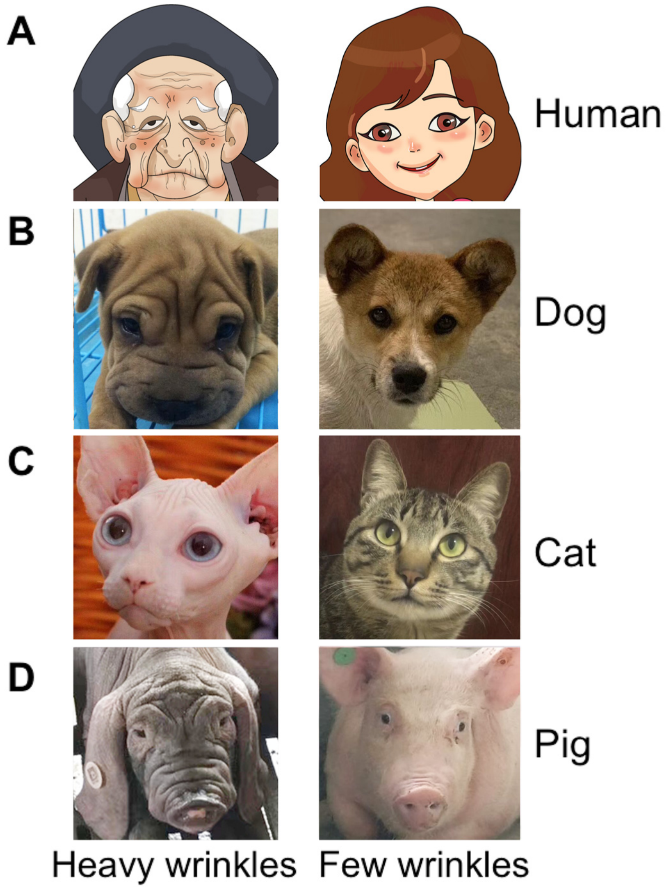

Figure S1. Comparison of heavy and few facial wrinkles in different species. (**A**) human. The screenshot of male head was captured from a cartoon grandpa with a hat; the screenshot of female head was captured from a cartoon lady with brown hair. (**B**) dog. Left head was from a Chinese Shar-pei dog; right head was from a Chinese Tianyuan dog. (**C**) cat. Left head was from a Canadian Sphynx cat; right head was from a Chinese Lihua cat. And (**D**) Pig. Left head was from a Chinese Erhualian pig; right head was from a Large White pig.

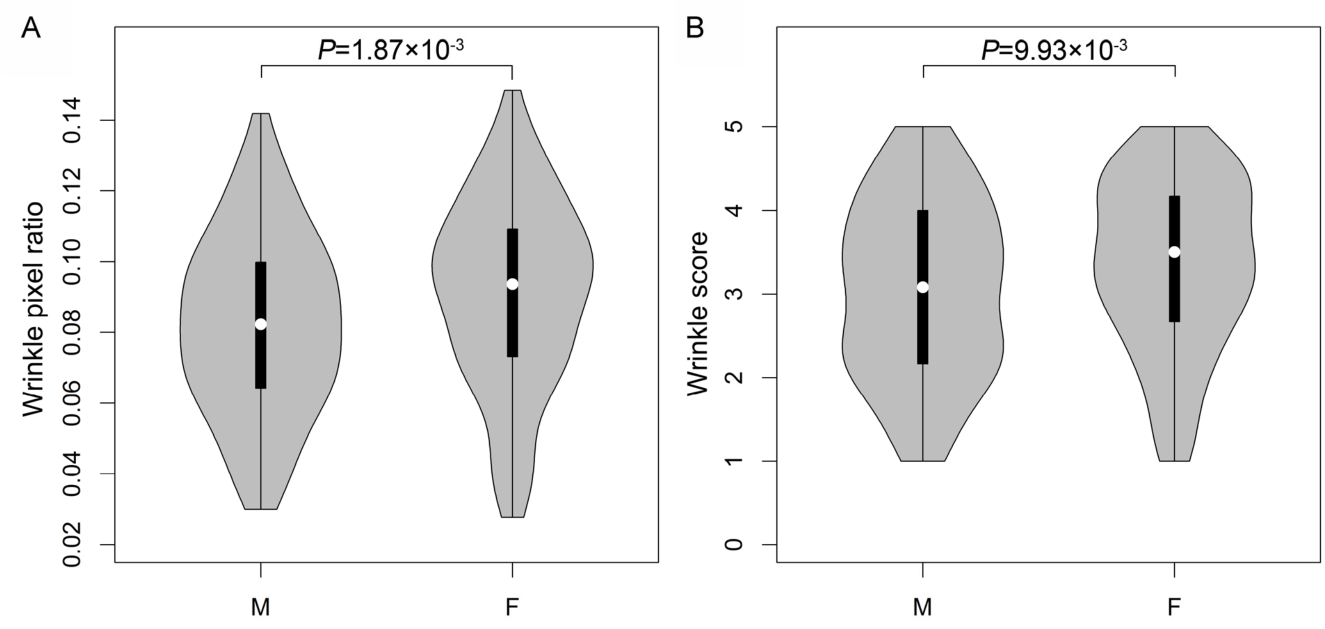

Figure S2. The difference of facial wrinkles between females and males.

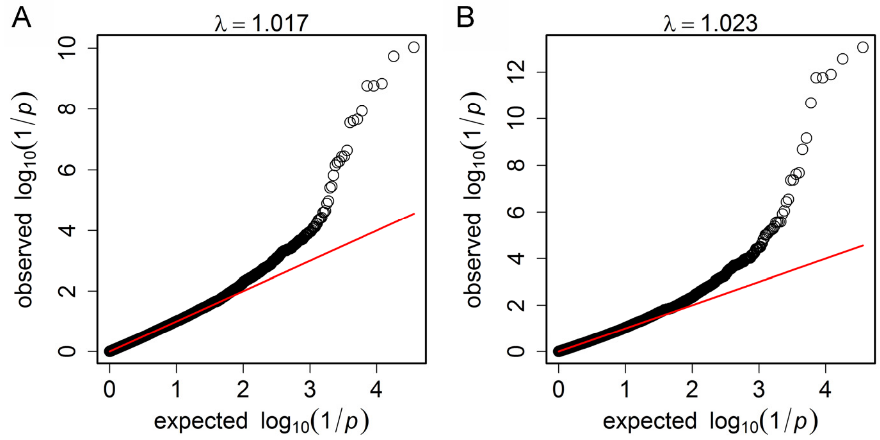

Figure S3. QQ plots for the GWAS of facial wrinkles. (A) wrinkle pixel ratio. (B) Winkle score.

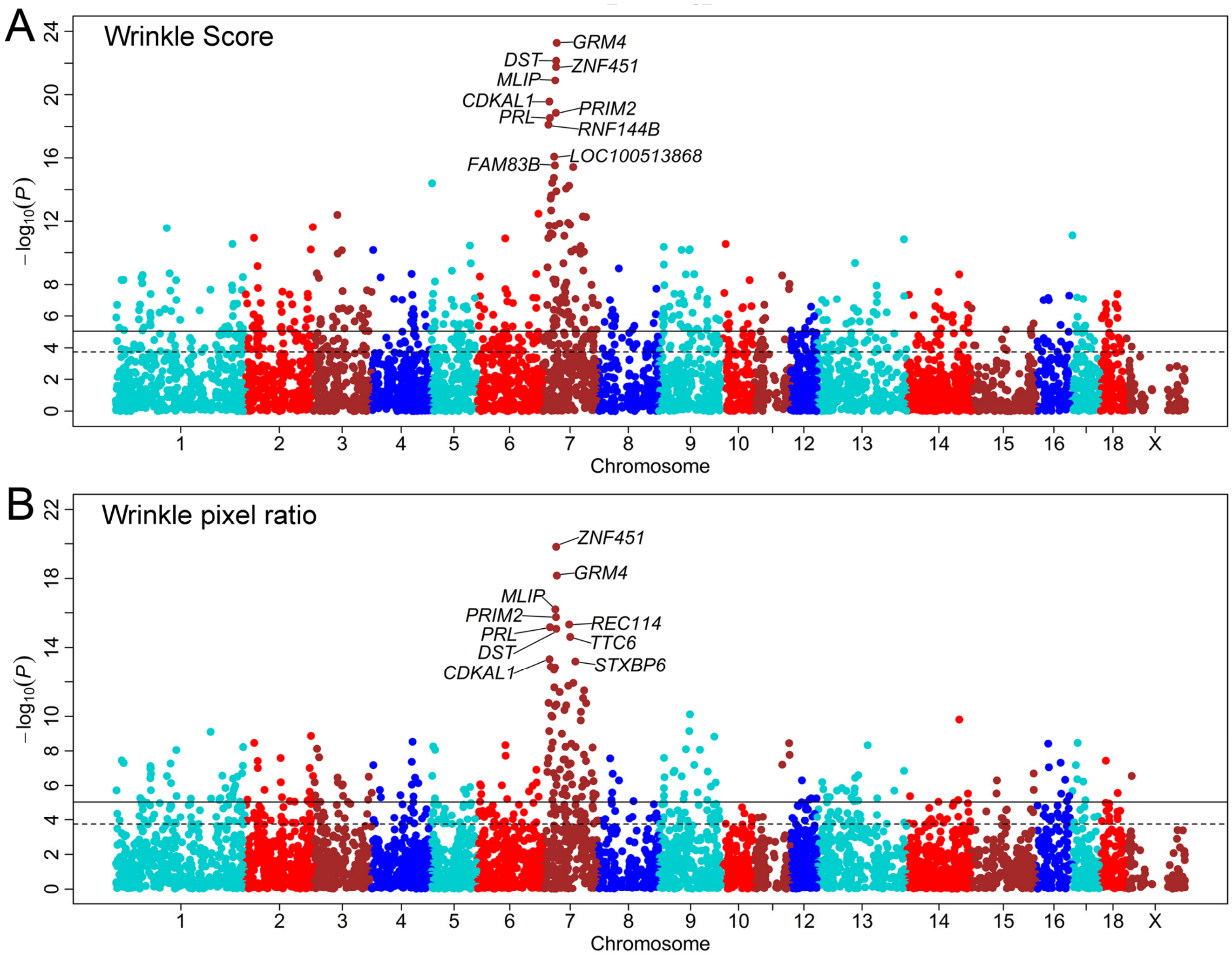

Figure S4. Genome-wide gene association analysis for facial wrinkles. (**A**) wrinkle pixel ratio. (**B**) Winkle score. The dashed line delineates the suggestive significance threshold; the solid line delineates the genome-wide significant threshold.

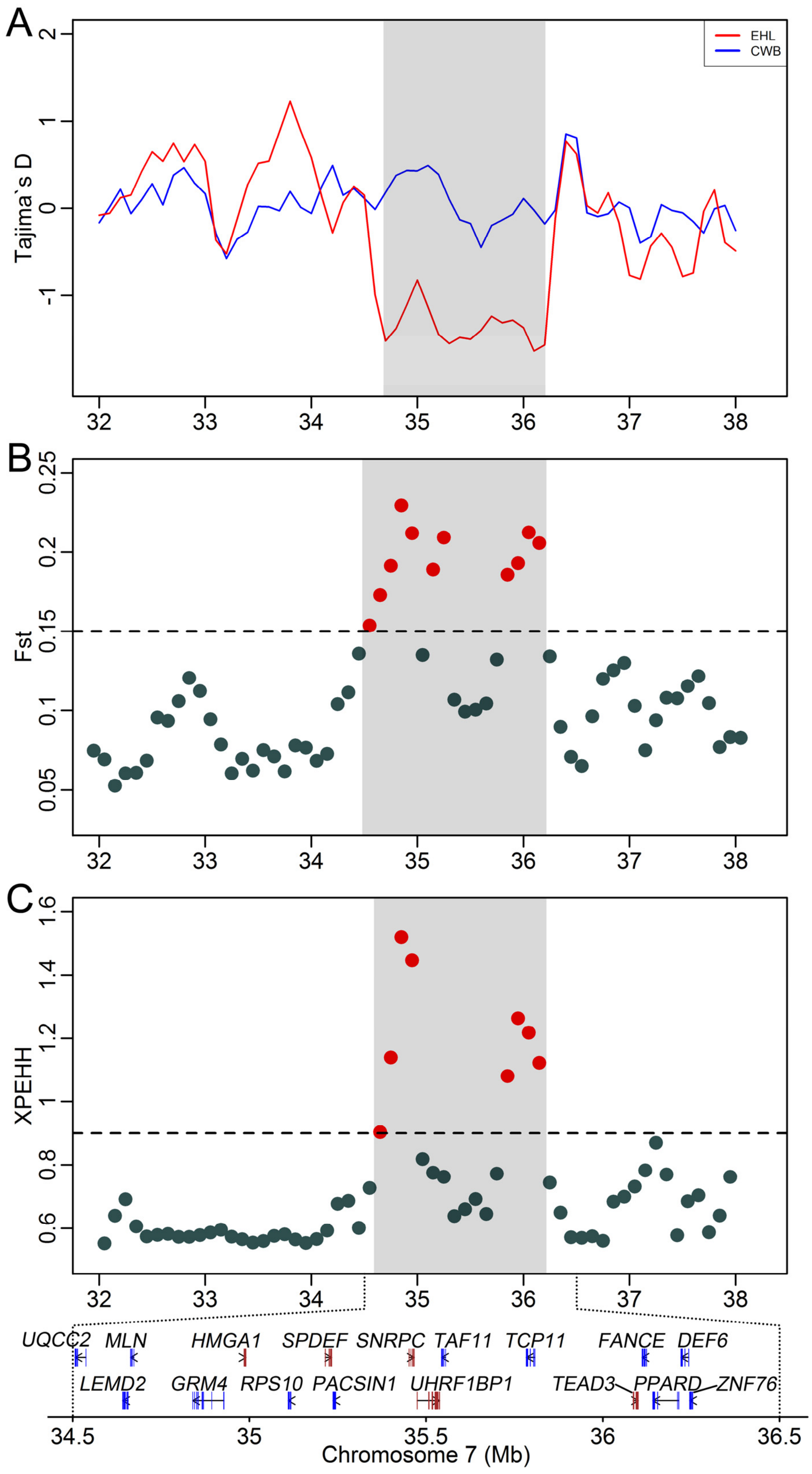

Figure S5. Evolutionary and selection analyses on the candidate region affecting swine facial wrinkles using the whole-genome sequencing data. (**A**) Tajima's D tests. (**B**) Population differentiation analysis between Erhualian pigs and Chinese wild boars. (**C**) XPEHH score estimation.

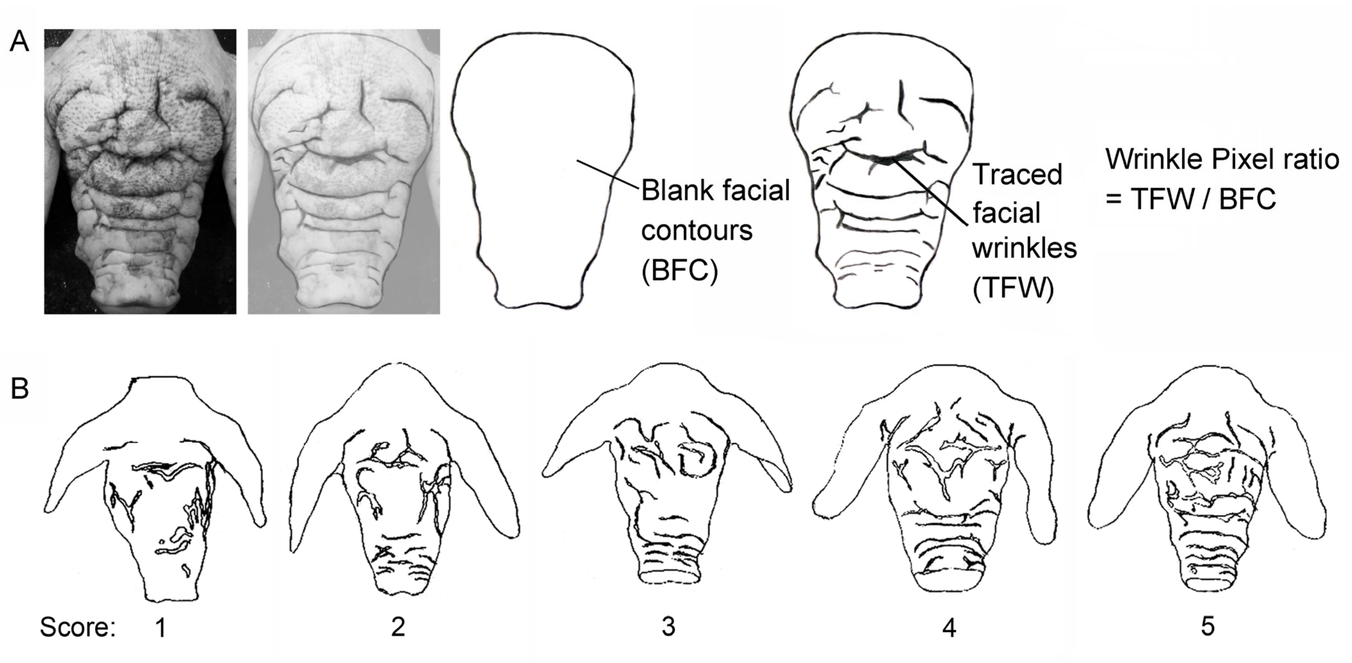

Figure S6. Two traits of swine facial wrinkles. (**A**) Estimation of wrinkle pixel ratio. (**B**) The criterion panel of facial wrinkle score.
